# Self management strategies for hypertensive patients on treatment in selected clinics at Ga-Dikgale, Capricorn District, Limpopo, South Africa

**DOI:** 10.3389/fpubh.2026.1872496

**Published:** 2026-08-21

**Authors:** Mamare Adelaide Bopape, Salphy Mamoropo Mmusi, Tebogo maria Mothiba

**Affiliations:** 1 Department of Nursing Science, University of Limpopo, Polokwane, South Africa; 2 National Research Foundation, SARChi, Faculty of Health Sciences, University of Limpopo, Polokwane, South Africa

**Keywords:** hypertension, patient education, primary healthcare, self management, South Africa, treatment adherence

## Abstract

**Background:**

Hypertension remains a major global public health challenge, with high levels of undiagnosed and poorly controlled disease contributing substantially to morbidity and mortality. Effective self management is critical for achieving optimal blood pressure control, particularly in resource constrained settings where healthcare access and continuity of care may be limited.

**Objective:**

To explore self management strategies among hypertensive patients receiving treatment at primary healthcare clinics in Ga-Dikgale, Capricorn District, Limpopo Province, South Africa.

**Methods:**

A qualitative, exploratory, descriptive, and contextual research design was employed. Data were collected through in-depth individual interviews with hypertensive patients and analyzed using Tesch's open coding method of thematic analysis.

**Results:**

Four themes emerged: (1) hypertension self management practices; (2) knowledge and understanding of hypertension management; (3) challenges affecting treatment adherence and blood pressure control; and (4) support systems for self-management. The findings revealed gaps between patients' knowledge and the implementation of recommended self-management behaviors, inconsistent medication adherence, reliance on both biomedical and traditional health practices, and the important influence of family support and healthcare system factors on hypertension management.

**Conclusion:**

Hypertension self management is shaped by complex interactions between individual behaviors, social support networks, and healthcare system factors. Strengthening patient centered education, ensuring consistent availability of antihypertensive medication, and enhancing family and community based support are essential strategies for improving hypertension control in rural primary healthcare settings.

## 1 Introduction

Hypertension remains a major global public health concern, contributing to approximately 7.5 million deaths annually, representing 12.8% of all deaths worldwide (1). It is the leading risk factor for premature mortality and is strongly associated with cardiovascular diseases, substantially contributing to early morbidity and mortality (2, 3). Poorly controlled hypertension significantly increases the risk of severe complications, including myocardial infarction, heart failure, stroke, and chronic kidney disease (4). Identifying individuals who are at risk but remain unaware of their hypertensive status or are not receiving appropriate treatment continues to be a major public health challenge (5).

Current evidence suggests that effective hypertension management depends not only on pharmacological treatment but also on patients' ability to engage in sustained self management behaviors, including adherence to prescribed medications, dietary modification, regular physical activity, weight management, smoking cessation, and moderation of alcohol consumption (6). Medication adherence remains a critical determinant of blood pressure control; however, adherence to both pharmacological and lifestyle recommendations is frequently suboptimal, particularly in low and middle income countries (LMICs), where health literacy limitations, socioeconomic challenges, and healthcare system constraints influence self management practices (7). Furthermore, patients' knowledge, health education exposure, cultural beliefs, and use of complementary or traditional remedies may shape hypertension management behaviors, highlighting the need for contextually appropriate interventions that strengthen self management and improve long term blood pressure outcomes (8).

Flynn reported that African American populations in the United States experience poorer hypertension control compared with White populations. The study further highlighted that effective hypertension management relies on key strategies, including medication adherence, regular blood pressure monitoring, and lifestyle modification, all of which contribute significantly to improved blood pressure control (9). Poor self management behaviors are recognized as important contributors to uncontrolled hypertension, while effective self management can enhance disease control, particularly among older adults who carry a greater burden of hypertension related complications (3). Flynn also emphasized the limited exploration of family members' perspectives regarding factors that facilitate or hinder their ability to support individuals living with hypertension (9).

In Ga-Dikgale primary healthcare clinics, many hypertensive patients continue to experience uncontrolled blood pressure despite receiving treatment. Among 444 patients receiving antihypertensive therapy in early 2018, 58, 67, and 70 patients had uncontrolled blood pressure in January, February, and March, respectively. Additionally, several patients were ineligible for the Centralized Chronic Medicines Dispensing and Distribution (CCMDD) programme, an initiative designed to improve access to chronic medication. These challenges highlight the need to explore how hypertensive patients manage their condition within this rural primary healthcare context.

Researchers from Wits University and their collaborators have reported that South Africa has the highest prevalence of hypertension in Southern Africa and the largest number of individuals with uncontrolled blood pressure despite treatment (10). Addressing this burden requires a patient centered chronic care approach that empowers individuals through improved knowledge, motivation, and behavior change skills (11). Effective chronic disease management should also incorporate an integrated, lifelong approach involving patients, families, communities, and healthcare providers (12).

Despite these efforts, the everyday experiences of people living with hypertension particularly how they interpret health education, adhere to long term medication regimens, and incorporate lifestyle modifications within resource constrained primary healthcare environments remain insufficiently explored in rural South African settings (13). Although South Africa has implemented national strategies to strengthen non communicable disease management through primary healthcare services, evidence indicates persistently high levels of uncontrolled hypertension (14). Limited qualitative research has examined how rural patients navigate structural barriers, including medication shortages in public healthcare facilities, unemployment, limited health literacy, and restricted access to healthcare services, all of which are recognized determinants of poor chronic disease outcomes in low resource settings (15).

Furthermore, cultural beliefs and the use of traditional or complementary medicines continue to influence health seeking behaviors and may affect adherence to biomedical hypertension treatment in South African communities (16). There remains insufficient qualitative understanding of how family and community support systems interact with individual knowledge, cultural practices, and healthcare system constraints to influence hypertension self management behaviors among rural populations, despite evidence demonstrating the important role of social support in chronic disease management (17).

Hypertension remains highly prevalent, with inadequate control persisting despite the availability of effective treatment, particularly in rural communities. This study addresses an important gap by exploring patient self management practices within a low-resource context, including medication adherence, lifestyle behaviors, and the integration of traditional remedies. By generating context specific qualitative evidence, the study aims to inform culturally appropriate and patient centered interventions to strengthen hypertension management and improve blood pressure control in rural primary healthcare settings (17).

## 2 Methods

### 2.1 Study design

An exploratory, descriptive, and contextual qualitative research design was employed to explore self management strategies among patients living with hypertension. This design was appropriate as it enabled an in depth understanding of participants' experiences, perceptions, and practices related to hypertension self management within their specific healthcare and social context.

### 2.2 Study site

The study was conducted at three primary healthcare clinics in Ga-Dikgale, Capricorn District, Limpopo Province, South Africa: Sebayeng Clinic, Seobi-Dikgale Clinic, and Dikgale Clinic. These clinics provide comprehensive primary healthcare services, including the diagnosis, treatment, and ongoing management of patients with chronic conditions such as hypertension.

### 2.3 Population and sampling

The study population comprised adults diagnosed with hypertension who had been receiving antihypertensive treatment for more than 6 months. Participants were eligible if they were aged 18 years or older, had a confirmed diagnosis of hypertension, and had experience managing their condition while receiving treatment. Patients who had received antihypertensive treatment for < 6 months or who were not currently receiving treatment were excluded, as they were considered unlikely to have sufficient experience with long-term hypertension self management practices.

Following ethical approval from the University Research Ethics Committee and permission from the Limpopo Department of Health, approval was obtained from the management of the participating clinics to recruit eligible participants. Potential participants were informed about the study objectives, procedures, potential risks and benefits, confidentiality measures, and their right to voluntarily participate or withdraw at any stage without consequences. Written informed consent was obtained from all participants before data collection commenced.

Eighteen eligible hypertensive patients were approached, of whom three declined participation due to time constraints or personal reasons. Fifteen participants provided written informed consent and completed the interviews, yielding a participation rate of 83.3%. Purposive sampling was used, and recruitment continued until data saturation was reached, as subsequent interviews generated no new codes or themes.

### 2.4 Data collection

Data were collected through in depth individual interviews with hypertensive patients using a semi structured interview guide consisting of open ended questions and probing prompts. Before the main study, a pilot study was conducted at one primary healthcare clinic to pre test the interview guide and assess the feasibility of the data collection procedures. Five purposively selected hypertensive patients who met the inclusion criteria participated in the pilot interviews. The interviews were audio recorded, transcribed verbatim, and reviewed by qualitative research experts to assess the clarity, relevance, and comprehensiveness of the interview guide. Based on expert feedback, minor modifications were made, mainly involving the refinement and rephrasing of probing questions to enhance clarity and facilitate the generation of richer and more detailed responses. The pilot study confirmed the appropriateness of the interview guide and the feasibility of the proposed data collection process. To minimize potential bias and avoid contamination of the main study findings, the pilot study site and participants were excluded from the final study sample.

Data collection for the main study was conducted in a language preferred and well understood by the participants and at venues convenient to them during their scheduled clinic visits. Interviews were conducted using the revised semi structured interview guide, which ensured consistent coverage of the research objectives while allowing flexibility for participants to describe their experiences, perceptions, and self management practices in depth. Prior to each interview, participants were informed about the use of audio recording equipment, and written informed consent was obtained. Participants were assured of confidentiality and informed of their right to withdraw from the study at any stage without any negative consequences.

With participants' permission, the researcher used field notes and a digital audio recorder to capture the interviews. Probing techniques were used throughout the interviews to explore responses further, clarify participants' views, and obtain detailed descriptions of their experiences with hypertension self management. Interviews were conducted in a private and quiet room free from interruptions and lasted approximately 30–40 min. Each interview commenced with the central question: “*Can you tell me about the self management strategies you use to control your blood pressure?”* Follow up questions and probing prompts were used to explore emerging issues and gain a comprehensive understanding of participants' experiences.

Data collection continued until saturation was achieved during the 11th interview, when no new concepts or themes emerged from the data. However, four additional interviews were conducted to confirm saturation, and these interviews yielded no new information. Therefore, data saturation was considered to have been achieved after 15 interviews.

### 2.5 Data analysis

The interviews were transcribed verbatim by the first author shortly after data collection to maintain contextual familiarity and facilitate immersion in the data. The transcripts were analyzed using Tesch's inductive descriptive coding technique, as described by Creswell (18). The first author repeatedly read and reviewed the transcripts to achieve data familiarization, identify significant statements, generate initial codes, and develop emerging patterns and potential themes.

An iterative coding process was followed, whereby codes were continuously compared, refined, and grouped into categories that reflected participants' experiences of hypertension self management. Both inductive and deductive coding approaches were applied; while codes and themes were primarily derived from participants' narratives, the research objectives and relevant theoretical concepts informed the interpretation and organization of the findings. Semantic coding was used to capture participants' explicit meanings and ensure that the identified themes remained grounded in their lived experiences.

To enhance the credibility and trustworthiness of the findings, the transcripts, audio recordings, coding framework, and emerging themes were independently reviewed by qualitative research experts. Discrepancies and areas of uncertainty were discussed and resolved through consensus. The identified themes were subsequently reviewed, refined, defined, and named to ensure coherence and alignment with the research objectives before the final interpretation and reporting of findings.

The analytical process is summarized in Table 1. The analysis generated four themes and corresponding sub themes describing self management practices among hypertensive patients attending primary healthcare clinics in Ga-Dikgale. The findings were interpreted in relation to existing literature and guided by Orem's Self-Care Theory.

**Table 1:** Steps of Tesch's open coding process.

Steps	Application to the study
Familiarizing with the data	The first author transcribed interviews verbatim and repeatedly reviewed the recordings and transcripts to gain an in-depth understanding of participants' experiences.
Identification of meaningful units	Meaningful text segments related to participants' experiences, perceptions, and coping strategies were identified and extracted.
Generation of initial codes	Transcripts were systematically coded line by line to identify concepts relevant to the research objectives.
Development of categories	Related codes were grouped into categories to identify patterns and develop preliminary themes and sub-themes.
Searching for themes	Categories were examined to identify recurring patterns and develop themes representing participants' experiences.
Reviewing and refining themes	Themes were reviewed against the coded data and full transcripts to ensure coherence, relevance, and accurate representation of the findings.
Defining and naming themes	Themes were refined, clearly defined, and named according to their central meanings, supported by relevant participant quotations.
Developing the final report	Final themes and sub-themes were presented as a coherent narrative, supported by participant quotations and reviewed by an independent qualitative expert to enhance trustworthiness.

### 2.6 Ensuring trustworthiness

Trustworthiness was ensured using Lincoln and Guba's four criteria: credibility, dependability, confirmability, and transferability.

*Credibility* was enhanced through prolonged engagement with participants, the use of semi structured interviews incorporating open ended questions and probing prompts, and conducting interviews in a private and comfortable environment that facilitated open communication. Prior engagement with clinic operational managers supported appropriate participant recruitment and ensured that data collection was conducted in accordance with chronic care appointment schedules. Member checking was conducted at the end of each interview by summarizing the researcher's understanding of participants' responses and interpretations. Participants were invited to confirm whether the summaries accurately represented their experiences, and any additional clarifications were incorporated into the final analysis.

*Dependability and confirmability* were strengthened through the maintenance of a comprehensive audit trail documenting all stages of the research process, including participant recruitment, interview procedures, field notes, reflexive notes, coding decisions, theme development, and data analysis. An independent qualitative researcher reviewed the coding process, analytical decisions, and emerging themes to enhance consistency, transparency, and methodological rigor. *Methodological triangulation* was achieved through the integration of semi structured interviews and field notes, supported by ongoing consultation with experienced qualitative researchers to enhance the credibility, consistency, and transparency of the analytical process.

Researcher bias was minimized through reflexivity, whereby the researcher critically reflected on personal assumptions, experiences, and potential influences throughout data collection and analysis. In addition, the use of a centralized interview guide with probing prompts and avoidance of leading questions helped ensure that participants' perspectives, rather than researcher expectations, guided the findings. *Transferability* was supported through the provision of a detailed description of the study context, participant characteristics, sampling strategy, data collection procedures, and analytical process. These descriptions enable readers to assess the relevance and applicability of the findings to comparable rural primary healthcare settings.

## 3 Results

### 3.1 Demographic profile of the participants

Table 2 presents the demographic characteristics of the 15 participants who took part in the study. The table summarizes the participants according to age, gender, marital status, and ethnic group, providing contextual information about the study sample.

**Table 2:** Participants' demographic details.

Characteristics	Number (15)	Percentage
**1.Age range in years**		
25–35	01	6%
36–45	04	27%
>45 years	10	67%
**2.Gender**		
Males	04	26%
Females	11	73%
**3.Marital Status**		
Single	03	20%
Married	09	60%
Divorced	00	0%
Widowed	03	20%
**4.Ethnic Group**		
Pedi	15	100%
Others	00	0%

### 3.2 Main findings

Four main themes and 12 corresponding sub themes emerged from the data analysis, capturing the self management strategies, experiences, and contextual factors influencing hypertension management among patients receiving treatment at selected primary healthcare clinics in Ga-Dikgale. These themes and their associated sub-themes are presented in Table 3.

**Table 3:** Themes and sub-themes.

Themes	subthemes
1.Hypertension self-management practices	1.1.Medication adherence
	1.2.Healthcare guidance for self management
	1.3.Dietary modification
	1.4.Physical activity
	1.5.Use of traditional and home remedies
2.Knowledge of hypertension self management	2.1.Knowledge of medication use
	2.2.Dietary knowledge
	2.3.Understanding the importance of medication
	2.4.Hypertension as a lifelong condition
3.Barriers to hypertension self management	3.1.Medication shortages
	3.2.Employment and financial challenges
	3.3.Uncertainty about persistent symptoms
4. Social Support for hypertension self management	4.1. Family support for self management

#### 3.2.1 Theme 1: Description of existing self management strategies by hypertensive patients on treatment

Participants described a range of self management strategies used to control their blood pressure. These included adherence to prescribed antihypertensive medication, compliance with dietary recommendations, engagement in physical activity, and the use of traditional or home remedies. Although most participants demonstrated awareness of recommended self management practices, their narratives revealed considerable variation in the extent to which these strategies were consistently implemented.

##### 3.2.1.1 Sub-theme 1.1: Medication adherence as a self management strategy

Participants generally recognized the importance of taking antihypertensive medication as prescribed. However, adherence was inconsistent, with some participants reporting occasional missed doses because of forgetfulness, whereas others described strict adherence to the prescribed regimen.

One participant acknowledged inconsistent medication adherence:

They give me pills, yes, I do take them when I remember so that my BP goes down a bit. But someone told me not to eat salty and oily foods; even at the clinic they told us to reduce those. (P9)

In contrast, another participant viewed antihypertensive medication as a means of prolonging life rather than curing the condition but nevertheless reported adhering to treatment instructions:

The medications that you give us just prolong our lives. They postpone death instead of dying this week, one will die next week (laughs). I do take them the way the sister told me. (P11)

These narratives demonstrate that while participants recognized the importance of antihypertensive medication, adherence behaviors varied, reflecting differences in beliefs, routines, and individual self management practices.

##### 3.2.1.2 Sub-theme 1.2. Healthcare guidance supporting self management

Participants consistently reported receiving health education from nurses during routine clinic visits. They described advice on taking medication at the same time each day, reducing dietary salt and fat intake, and engaging in regular physical activity. Many participants indicated that these recommendations informed their daily self management practices.

One participant explained:

I take my medication because the sister at the clinic told me that if I take it at the same time every day, my blood pressure will be okay. (P4)

Another participant similarly reported:

I just take these pills, and since they told us that we shouldn't eat salt and oil, I follow that. The sisters at the clinic told me to take my medication as instructed. (P5)

Likewise, another participant stated:

I take the medication at the same time as the sister told me. (P7)

These findings suggest that healthcare professionals played an important role in promoting hypertension self-management, with participants frequently recalling and attempting to implement the advice received during clinic consultations.

##### 3.2.1.3 Sub-theme 1.3: Dietary modification for blood pressure control

Dietary modification was one of the most frequently reported self management strategies. Participants commonly described reducing their intake of salt and fatty foods following advice received from healthcare providers. However, few participants referred to broader dietary recommendations such as increasing fruit, vegetable, or whole-grain consumption.

One participant stated:

I don't take a lot of salt, I don't take sugar. I was advised and educated about the importance of reducing salt and fatty foods in the diet. (P2)

Another participant explained:

Regarding salt and oil, they tell us here at the clinic to reduce them. (P3)

Some participants described adopting practical alternatives to reduce salt consumption:

I use alternatives like spices so that I can leave out salt; that is the better way. (P6)

These accounts indicate that participants primarily associated dietary self management with limiting salt and fat intake, reflecting the emphasis placed on these recommendations during health education.

##### 3.2.1.4 Sub-theme 1.4: Physical activity as a self management strategy

Participants acknowledged the importance of physical activity in controlling hypertension. Rather than engaging in structured exercise programmes, most described routine daily activities such as walking, farming, gardening, and household chores as their primary forms of exercise.

One participant explained:

I am the kind of person who keeps walking since I am a herder. I exercise by walking all day long. Yes, it helps me because I become a little better, but at other times my HBP goes up and down. I also drink 2 L of water daily. (P2)

Another participant reported:

They once told us at the clinic to walk for about an hour. I also work around the yard and in the field. At times I have more energy because walking around the village allows me to see other people. At home, there is nothing much I do, as I am a pensioner. (P3)

However, some participants reported physical limitations that restricted their ability to exercise. One participant stated:

I wake up from bed. However, this leg is broken (fracture), so I cannot bend it. I just stretch my hands and legs while lying on the bed. (P4)

Another explained:

Regarding exercise, I was advised, but I do not have time because I have joint pain. (P13)

These findings illustrate that although participants recognized the value of physical activity, engagement was influenced by age, mobility limitations, chronic pain, occupational responsibilities, and functional ability.

##### 3.2.1.5 Sub-theme 1.5: Use of traditional and home remedies in hypertension management

Many participants reported using traditional or home remedies alongside prescribed antihypertensive medication. Frequently mentioned remedies included moringa, boiled water (“Mogamolo”), and indigenous medicinal plants. Participants generally perceived these remedies as complementary rather than substitutes for prescribed treatment.

One participant described combining moringa with prescribed medication:

I normally take my medication and also use moringa. It is a green powder (lerole). I sprinkle it on my food. Moringa makes a difference to my BP; when I do not take it, my BP goes up to around 160 mmHg. I take moringa with breakfast and then take my medication after one hour. I take them three times a day. I cannot say moringa affects my medication; I still take my medication as prescribed. I do not want to die (laughs). (P1)

Another participant stated:

Most of my help comes from the church. I drink water called 'Mogamolo.' I also take the clinic medication. 'Mogamolo' is just boiled water that has cooled to room temperature. (P6)

Similarly, another participant explained:

No, 'mokgadi wa sega sega' is a small plant that grows around morula trees. Moringa is a powder. I sometimes use moringa when I can afford it. 'Mokgadi wa sega sega' is always available in the bushes. (P13)

These accounts demonstrate that traditional and home remedies formed an integral component of participants' self management practices. Rather than replacing prescribed antihypertensive medication, these remedies were generally perceived as complementary approaches for maintaining blood pressure control.

#### 3.2.2 Theme 2: Description of existing knowledge among patients living with hypertension

This theme describes participants' understanding of hypertension and its management. Participants discussed their knowledge of antihypertensive medication, dietary recommendations, treatment adherence, and the chronic nature of hypertension. Although most participants demonstrated basic knowledge of hypertension management, important knowledge gaps remained regarding disease progression, comprehensive lifestyle modification, and long-term self-management.

##### 3.2.2.1 Sub-theme 2.1: Knowing when and how to take medication

Participants demonstrated varying levels of knowledge regarding their prescribed antihypertensive medication. While several participants accurately described the dosage, timing, and administration of their medication, others reported combining prescribed treatment with traditional or faith-based remedies as part of their medication taking routine.

One participant explained:

They all have different times. There is one I take once a day, and the other one I take three times a day. (P6)

Another participant described integrating prescribed medication with traditional remedies:

Ah, I don't skip my medication. These pills have their times. I drink the tea or leaves in the morning after breakfast, and then around 10 am I take my medication. (P7)

Similarly, another participant reported:

I take my medication in the morning, midday, and evening. But I take moringa in the morning and sometimes at midday. (P10)

Faith-based practices also influenced medication routines. One participant explained:

When you wake up in the morning, you use your small kettle. When the water becomes lukewarm, you drink it once or twice a day before taking your medication. These are church practices. They are things that we sleep and wake up with. (P11)

These accounts suggest that participants generally understood how and when to take their prescribed medication. However, medication practices were frequently shaped by cultural and faith based beliefs, with many participants integrating complementary remedies into their treatment routines.

##### 3.2.2.2 Sub-theme 2.2: Dietary knowledge shaped by health education

Participants demonstrated basic knowledge of dietary recommendations for hypertension management. Most recognized the importance of reducing salt and fat intake, largely reflecting health education received at primary healthcare clinics. However, few participants described broader dietary recommendations, such as increasing the consumption of fruits, vegetables, legumes, or whole grains. Dietary practices were also influenced by traditional eating habits, household food preferences, and available resources.

One participant described a traditional breakfast routine:

No (laughs), I usually take it in the morning with my soft porridge (smiles). I am an old person from the old days. Soft porridge is still our breakfast, not the food people eat nowadays. (P1)

Another participant explained:

They told us that reducing salt and oil in your food helps control blood pressure. Too much salt and oil increase your blood pressure. (P5)

Similarly, another participant stated:

They tell us to reduce salt and oil, and I follow those rules. I always do what they tell me because I know it helps me stay alive. (P6)

Another participant recalled receiving dietary counseling from healthcare professionals:

Firstly, I don't take a lot of salt. Secondly, I avoid eating too many oily foods. The nurses and the dietician explained that I must reduce salt and oily foods. (P13)

These findings indicate that participants commonly associated dietary management with reducing salt and fat intake, while demonstrating more limited knowledge of other evidence based dietary recommendations for hypertension management.

##### 3.2.2.3 Sub-theme 2.3: Medication as a means of preserving health and life

Participants generally recognized the importance of antihypertensive medication in maintaining health and preventing hypertension related complications. Medication was commonly viewed as essential for controlling blood pressure, improving well being, and prolonging life. Nevertheless, some participants continued to associate persistent symptoms with inadequate blood pressure control despite adherence to treatment.

One participant stated:

The treatment is working. I take my medication accordingly. I don't want to die (laughs). (P1)

Another participant reported:

Yes, they do help me because I become a little better, but at other times my HBP goes up and down. I also drink 2 L of water daily. (P2)

A third participant explained:

I take my treatment, and it helps. My main problem is that I often feel dizzy. I don't know what causes it. Whenever I become dizzy, I go into the clinic to have my vital signs checked, and they tell me that my blood pressure is well controlled. (P14)

These narratives demonstrate that participants understood the importance of medication in maintaining health. However, ongoing symptoms created uncertainty about disease control, highlighting potential gaps in patients' understanding of hypertension and symptom interpretation.

##### 3.2.2.4 Sub-theme 2.4: Recognizing hypertension as a lifelong responsibility

Participants demonstrated varying levels of understanding regarding hypertension as a lifelong chronic condition requiring continuous treatment and self management. While most recognized the need for regular clinic attendance and ongoing medication, several participants reported limited understanding of the disease itself and its long term consequences.

One participant explained:

No, I don't know much. I only know that I have high blood pressure and that I must go to the clinic. My blood pressure was around 200 mmHg, and when I came to the clinic, they started giving me medication. (P2)

Another participant indicated that most of their knowledge was acquired through health education sessions at the clinic:

I have a little information because they teach us how to take care of ourselves during health education at the clinic. Some days they talk about TB, diabetes, and other illnesses. That's the information I have. (P10)

Similarly, another participant stated:

I was told that I have high blood pressure, and I realized that it is my responsibility because I am the one living with this condition. Otherwise, the healthcare providers will say that my blood pressure is uncontrolled. (P13)

These findings suggest that participants generally recognized hypertension as a condition requiring lifelong treatment and regular follow up. However, their understanding of the underlying disease process, long term complications, and the rationale for sustained self management varied considerably.

#### 3.2.3 Theme 3: Barriers affecting hypertension self management among patients receiving treatment

Participants described multiple challenges that influenced their ability to effectively manage hypertension while receiving treatment. These challenges extended beyond individual self management behaviors and included health system related barriers, socioeconomic constraints, and uncertainties regarding hypertension symptoms and disease control. Participants highlighted how medication availability, employment difficulties, financial limitations, and persistent symptoms affected their ability to adhere to treatment recommendations and maintain confidence in their hypertension management.

##### 3.2.3.1 Sub-theme 3.1: Medication shortages as a barrier to blood pressure control

Participants reported a strong reliance on public healthcare facilities for their monthly antihypertensive medication. However, several participants described experiencing medication shortages during clinic visits, which disrupted continuity of treatment and created uncertainty regarding their ability to maintain adequate blood pressure control. Financial constraints further limited their ability to obtain medication from alternative sources, such as private pharmacies.

One participant stated:

I think my HBP can be better controlled when I find all my medications at the clinic during my visits because sometimes the nurses tell you that a particular medicine is out of stock. (P3)

Another participant explained:

Sometimes when you come to the clinic to collect your medication, you find that some medicines are out of stock. I understand that it is not the clinic's fault. Some patients who were waiting with me told me that moringa helps with high blood pressure. (P5)

Similarly, another participant reported:

I don't want to lie, Auntie. Since I was diagnosed, my hope is only in this clinic. But sometimes the medications are out of stock, and I don't have money to buy them or go to another clinic. (P11)

These accounts demonstrate that medication shortages were perceived as a significant barrier to consistent hypertension management. The dependence on public healthcare facilities meant that interruptions in medication supply directly influenced participants' confidence in achieving stable blood pressure control and, in some cases, encouraged consideration of alternative remedies.

##### 3.2.3.2 Sub-theme 3.2: Employment and financial challenges associated with hypertension

Participants described socioeconomic challenges associated with living with hypertension, particularly difficulties related to employment opportunities and financial security. Some participants perceived their hypertension diagnosis as a barrier to obtaining employment, especially where medical fitness assessments were required. Financial limitations also affected their ability to seek alternative healthcare services or purchase medication when it was unavailable at public facilities.

One participant stated:

Regarding work, employers do not hire us because I have this condition. (P2)

Another participant explained:

When I was looking for a job, the employer required us to undergo a medical fitness assessment. They found that I had high blood pressure and told me to go to the clinic. My blood pressure was around 200 mmHg, and that is when I started receiving treatment. (P9)

These experiences suggest that hypertension was not only perceived as a health concern but also as a condition with broader social and economic consequences. Participants' narratives highlight how socioeconomic vulnerability may reduce their capacity to engage consistently in long term hypertension management.

##### 3.2.3.3 Sub-theme 3.3: Uncertainty about the causes of persistent symptoms

Participants described uncertainty regarding the relationship between their symptoms and hypertension control. Some reported experiencing dizziness, headaches, and feelings of faintness despite being informed by healthcare providers that their blood pressure was controlled. Others expressed limited understanding of hypertension symptoms and reported that their diagnosis was unexpected because they had no previous awareness of the condition.

One participant stated:

My main problem is that I usually feel dizzy. I don't know what causes it. Whenever I become dizzy, I go into the clinic to have my vital signs checked, and they tell me that my blood pressure is well controlled. (P14)

Another participant explained:

I usually experience headaches, dizziness, and feel faint. At first, I thought it was diabetes, but the doctor told me that I do not have diabetes. (P5)

These accounts illustrate a gap between clinical assessments of blood pressure control and patients' lived experiences of symptoms. The uncertainty surrounding symptom interpretation may contribute to anxiety, self monitoring challenges, and difficulties in understanding the ongoing nature of hypertension as a chronic condition.

#### 3.2.4 Theme 4: Social support as a facilitator of hypertension self-management

Participants described receiving support from family members, friends, and healthcare providers, which contributed to their ability to manage hypertension. The support provided included reminders to take medication, attend scheduled clinic appointments, and follow recommended dietary practices. Among these sources of support, family members emerged as the primary source of day-to-day assistance, particularly in helping participants maintain treatment routines and overcome challenges related to limited literacy.

##### 3.2.4.1 Sub-theme 4.1: Family involvement in supporting hypertension self-management

Participants reported sharing their hypertension diagnosis with family members, who subsequently became actively involved in supporting their self management practices. Family members commonly assisted by reminding participants to take medication, attend clinic appointments, and adhere to dietary recommendations. For participants with limited literacy, family members also provided practical assistance by reading appointment dates and interpreting health-related information.

One participant explained:

I stay with my grandchildren, and they are already in higher grades. I am not sure which grade, but they help me because they remind me about my clinic appointment dates. Even when the sister writes in my clinic appointment book, I cannot read it, so they help me. (P1)

Another participant described family involvement in dietary practices and medication adherence:

My grandchildren do not add any salt to the food when they are cooking. They also remind me to take my medication. (P6)

Similarly, another participant stated:

They remind me when it is time to take my treatment and about the date of my clinic appointment. (P11)

These accounts demonstrate that family members played a critical role in strengthening hypertension self management by supporting medication adherence, facilitating clinic attendance, and assisting participants in implementing recommended lifestyle modifications.

## 4 Discussion

This study explored the self management practices, knowledge, challenges, and support systems of patients living with hypertension who were receiving treatment in primary healthcare settings. The findings demonstrate that hypertension self management is a multidimensional process shaped by the interaction between individual behaviors, health literacy, healthcare system factors, socioeconomic circumstances, cultural beliefs, and family support. Although participants demonstrated awareness of recommended hypertension management practices, important gaps existed between knowledge and implementation, highlighting the complexity of achieving optimal blood pressure control in resource-constrained settings.

From the perspective of Orem's Self-Care Theory, effective hypertension management requires individuals to possess adequate self care agency the ability to understand, initiate, and maintain behaviors necessary to manage their condition. Participants demonstrated elements of self-care agency through medication use, dietary modification, physical activity, and engagement with healthcare services. However, their ability to perform these activities was influenced by contextual factors, including limited health literacy, medication availability, socioeconomic challenges, and reliance on complementary health practices (19).

Self management requires active patient participation in controlling blood pressure through adherence to prescribed medication, appropriate dietary practices, physical activity, and sustained lifestyle modification (20). Similarly, self care among individuals with hypertension encompasses healthy dietary practices, regular physical activity, smoking cessation, avoidance of excessive alcohol consumption, weight management, and consistent medication adherence (21). As a chronic condition requiring lifelong management, hypertension depends on continuous engagement between patients and healthcare systems to maintain treatment behaviors over time (22). However, this study found that although healthcare providers emphasized correct medication use, some participants reported taking medication only when they remembered. This finding suggests that awareness of treatment recommendations does not necessarily translate into consistent adherence. Previous research has identified medication non adherence as a major contributor to uncontrolled hypertension and increased cardiovascular and cerebrovascular complications (23, 24). Therefore, hypertension management programmes should extend beyond medication provision by incorporating continuous adherence support, patient counseling, and follow up strategies that address the practical challenges patients experience in daily life.

Participants reported receiving lifestyle related advice from healthcare providers, particularly regarding salt reduction, avoidance of fatty foods, medication adherence, and physical activity (25). However, dietary practices were primarily focused on avoiding specific foods rather than adopting comprehensive dietary patterns recommended for cardiovascular health. Few participants discussed the importance of fruits, vegetables, and high fiber foods, suggesting that dietary education may not have fully addressed broader nutritional requirements. Similar findings have been reported by Atakle et al., who demonstrated that although patients often understand the relationship between salt intake and hypertension, knowledge of wider dietary recommendations remains limited (26). Given the established relationship between excessive sodium intake and elevated blood pressure, dietary counseling should move beyond individual food restrictions toward culturally appropriate guidance on sustainable healthy eating patterns.

Physical activity was recognized by participants as beneficial for blood pressure control; however, most relied on routine household activities, farming, or walking rather than structured exercise programmes. Physical limitations, aging, and competing responsibilities influenced their ability to engage in formal exercise. These findings align with evidence that regular physical activity contributes to improved blood pressure control and reduced cardiovascular risk (27, 28). In rural settings, interventions should therefore promote realistic and accessible forms of physical activity that align with patients' daily routines rather than relying solely on structured exercise recommendations that may not be feasible.

An important finding of this study was the reported use of traditional remedies alongside prescribed antihypertensive medication. Participants described using remedies such as Moringa oleifera as part of their personal approaches to managing hypertension while continuing with biomedical treatment. These accounts reflect the coexistence of traditional and conventional health practices within rural communities and illustrate how patients incorporate culturally familiar practices into their self management routines. Although some experimental studies have suggested potential biological properties of Moringa oleifera, current clinical evidence remains insufficient to support its use as an established antihypertensive therapy (29). The reported use of indigenous remedies may be influenced by factors such as accessibility, affordability, cultural beliefs, and perceptions regarding safety and fewer adverse effects compared with conventional medicines (30). These findings highlight the importance of culturally sensitive healthcare communication, where healthcare providers routinely enquire about complementary medicine use and provide appropriate guidance to support safe integration with prescribed treatment and minimize potential risks associated with herb drug interactions.

The study also identified important gaps in participants' understanding of hypertension. While participants recognized hypertension as a chronic condition requiring lifelong treatment, many had limited knowledge regarding disease causes, risk factors, and long term complications. Awareness was largely focused on medication use and reducing salt intake, with less recognition of other modifiable risk factors such as obesity, alcohol consumption, and physical inactivity. This reflects broader challenges in hypertension control in South Africa, where awareness, treatment, and control rates remain inadequate despite the substantial burden of disease (31, 32). Previous studies have demonstrated that improved hypertension knowledge contributes to better adherence, self care behaviors, and disease control (33). Strengthening patient education should therefore include comprehensive information about disease mechanisms, risk factors, treatment expectations, and prevention of complications.

Health literacy emerged as a key determinant influencing participants' ability to implement self management behaviors. Several participants experienced difficulty interpreting medication instructions and relied on tablet color, shape, or appearance rather than medication names or prescribed doses. This finding supports previous evidence demonstrating that inadequate health literacy affects medication understanding, adherence, and chronic disease self management (34–36). In rural primary healthcare settings, improving health literacy requires context-appropriate approaches, including simplified communication, visual medication aids, and repeated patient-centered education.

Knowledge of dietary sodium intake and its relationship with hypertension is an important determinant of patients' dietary behaviors. However, evidence suggests that adherence to dietary recommendations remains suboptimal unless nutrition counseling is explicitly incorporated into routine hypertension management (37). Misconceptions and limited understanding of hypertension and its nutritional requirements contribute to unhealthy lifestyle behaviors and inadequate blood pressure control (38). Similarly, adequate knowledge of hypertension and its treatment is fundamental to safe medication use, sustained adherence, and improved quality of life. Amer reported that patients with greater disease-related knowledge and more positive attitudes toward treatment are more likely to adhere to prescribed therapy and achieve better clinical outcomes (39). Conversely, inadequate understanding of hypertension has been associated with poor medication adherence and uncontrolled blood pressure (40). These findings reinforce the importance of comprehensive, patient-centered education that addresses both lifestyle modification and medication management to strengthen self management behaviors and improve long-term blood pressure control (41).

Medication availability was another important challenge affecting hypertension management. Participants reported experiencing shortages of antihypertensive medication at public healthcare facilities and indicated that financial limitations restricted their ability to obtain treatment elsewhere. Such interruptions may undermine adherence and contribute to unstable blood pressure control. Similar concerns have been documented internationally, where medicine shortages have been associated with treatment disruption, poorer clinical outcomes, and increased patient uncertainty (42, 43). Strengthening medicine supply systems remains essential for improving continuity of care and achieving hypertension control targets.

Socioeconomic circumstances emerged as an important contextual factor influencing participants' ability to manage hypertension. Some participants perceived their hypertension diagnosis as a barrier to employment opportunities, particularly when medical fitness assessments were required during recruitment processes. These experiences suggest that hypertension may extend beyond a clinical condition by influencing economic participation and livelihood security. In addition, financial constraints limited participants' ability to access alternative healthcare services or purchase medication when treatment was unavailable at public healthcare facilities. Similar evidence indicates that socioeconomic disadvantage, employment insecurity, and financial barriers negatively influence chronic disease management by restricting access to healthcare resources and reducing individuals' capacity to engage in recommended self management behaviors (44, 45). Although this study does not establish causal relationships between hypertension and unemployment, participants' narratives illustrate how social determinants of health interact with healthcare system challenges to shape experiences of living with hypertension. Therefore, hypertension management should extend beyond individual behavioral interventions to incorporate broader social and economic realities that influence patients' ability to achieve sustained disease control, particularly in resource constrained settings.

Family support emerged as an important facilitator of hypertension self management. Participants described family members assisting with medication reminders, clinic appointments, dietary choices, and interpretation of written information. This support was particularly valuable among participants with limited literacy. Previous studies have similarly demonstrated that family involvement enhances treatment adherence, encourages healthy behaviors, and contributes to improved hypertension outcomes (46, 47). Integrating family members into hypertension education and care planning may therefore strengthen long-term self-management, particularly in rural communities where family networks remain central to daily support.

Overall, this study demonstrates that hypertension self management extends beyond individual responsibility and is shaped by interconnected personal, social, cultural, and healthcare system factors. Guided by Orem's Self Care Deficit Nursing Theory, the findings highlight that effective hypertension management requires patients' ability to acquire knowledge, make informed decisions, and engage in health promoting behaviors, while recognizing that contextual barriers may limit their capacity to fulfill self-care needs. These barriers included limited health literacy, medication access challenges, socioeconomic constraints, and uncertainty regarding disease management. Therefore, nursing and primary healthcare interventions should focus on enhancing patients' capacity for self management through tailored health education, supportive counseling, culturally sensitive communication, reliable access to treatment, and active family involvement. This approach can promote patient centered hypertension care that empowers individuals to participate effectively in long term disease control while ensuring appropriate support within resource constrained settings.

## 5 Limitations of the study

This study was conducted among hypertensive patients receiving treatment at selected primary healthcare clinics in Ga-Dikgale village, Capricorn District, Limpopo Province, South Africa. The context specific nature of the study setting may limit the transferability of the findings to other healthcare contexts, geographical regions, or populations with different sociocultural and healthcare characteristics. However, the detailed description of the study context, participants, sampling approach, data collection procedures, and analytical process enables readers to assess the applicability of the findings to similar settings.

The study involved a relatively small sample size of 15 participants recruited through purposive sampling, which may have limited the diversity of perspectives captured. However, recruitment continued until data saturation was achieved, with no new themes emerging from subsequent interviews, supporting the adequacy of the sample for the qualitative design. As participants were selected based on specific eligibility criteria and their ability to provide relevant experiences, the findings should be interpreted as contextually grounded rather than statistically representative.

The study relied on participants' self reported experiences, which may have been influenced by recall bias and social desirability bias. Participants may have unintentionally omitted certain experiences or provided responses perceived as acceptable, particularly regarding medication adherence, lifestyle practices, and interactions with healthcare providers. To minimize these potential biases, the researcher used a semi-structured interview guide with probing questions, conducted interviews in a private setting, and applied reflexive practices throughout data collection and analysis.

Although the study provides valuable insights into the complex and multidimensional nature of hypertension self management, the findings should be understood within the context in which they were generated. Participants' experiences were shaped by individual knowledge, health literacy, healthcare system constraints, socioeconomic circumstances, cultural beliefs, and family support. Further research involving diverse geographical locations, larger participant groups, and varied healthcare settings is recommended to strengthen the evidence base and inform contextually appropriate interventions for hypertension self management.

## 6 Conclusion

Hypertension self management in this rural South African setting is a complex process shaped by the interaction of individual, social, and healthcare system factors. Although participants demonstrated awareness of some recommended self management practices, gaps remained in their understanding of hypertension, lifestyle modification, and long term disease management, contributing to challenges in achieving optimal blood pressure control.

The findings highlight that effective hypertension management extends beyond individual patient behavior and is influenced by health literacy, continuity of medication supply, socioeconomic circumstances, cultural health practices, and family support. Family involvement emerged as an important resource that strengthened medication adherence, clinic attendance, and dietary practices, particularly among participants experiencing literacy related challenges.

These findings emphasize the need for patient centered, contextually appropriate interventions that strengthen health literacy, promote informed selfcare decisions, and address misconceptions surrounding hypertension management. Strengthening primary healthcare services through reliable medication availability, improved patient education, and meaningful engagement of families and communities is essential for improving long-term hypertension outcomes in rural settings.

## Data Availability

The raw data supporting the conclusions of this article will be made available by the authors, without undue reservation.
